# Opportunities to improve quality of care for cancer survivors in primary care: findings from the BETTER WISE study

**DOI:** 10.1007/s00520-023-07883-4

**Published:** 2023-06-30

**Authors:** Aisha Lofters, Ielaf Khalil, Nicolette Sopcak, Melissa Shea-Budgell, Christopher Meaney, Carolina Fernandes, Rahim Moineddin, Denise Campbell-Scherer, Kris Aubrey-Bassler, Donna Patricia Manca, Eva Grunfeld

**Affiliations:** 1 Department of Family and Community Medicine, University of Toronto, 500 University Ave, Toronto, Ontario M5G 1V7 Canada; 2 Peter Gilgan Centre for Women’s Cancers, Women’s College Hospital, 76 Grenville St, Toronto, ON M5S 1B2 Canada; 3 Sinai Health, 600 University Ave, Toronto, Canada; 4 Charbonneau Cancer Institute and Department of Oncology, University of Calgary, 3280 Hospital Drive NW, Calgary, Alberta T2N 4Z6 Canada; 5 Department of Family Medicine, University of Alberta, 6-10 University Terrace, Edmonton, Alberta T6G 2T4 Canada; 6 Office of Lifelong Learning & Physician Learning Program, University of Alberta, 2-590 Edmonton Clinic Health Academy, Edmonton, Alberta T6G 1C9 Canada; 7 Discipline of Family Medicine, Memorial University of Newfoundland, 300 Prince Phillip Drive, St. John’s, Newfoundland A1B 3V6 Canada; 8 Covenant Health, Grey Nuns Community Hospital, 1100 Youville Drive Northwest, Edmonton, Alberta T6L 5X8 Canada; 9 Ontario Institute for Cancer Research, 661 University Avenue, Suite 510, Toronto, ON M5G 0A3 Canada

**Keywords:** Primary care, Cancer survivorship, Survivorship guidelines, Pragmatic trial

## Abstract

**Purpose:**

The BETTER WISE (Building on Existing Tools to Improve Chronic Disease Prevention and Screening in Primary Care for Wellness of Cancer Survivors and Patients) intervention is an evidence-based approach to prevention and screening for cancers and chronic diseases in primary care that also includes comprehensive follow-up for breast, prostate and colorectal cancer survivors. We describe the process of harmonizing cancer survivorship guidelines to create a BETTER WISE cancer surveillance algorithm and describe both the quantitative and qualitative findings for BETTER WISE participants who were breast, prostate or colorectal cancer survivors. We describe the results in the context of the COVID-19 pandemic.

**Methods:**

We reviewed high-quality survivorship guidelines to create a cancer surveillance algorithm. We conducted a cluster randomized trial in three Canadian provinces with two composite index outcome measured 12 months after baseline, and also collected qualitative feedback on the intervention.

**Results:**

There were 80 cancer survivors for whom we had baseline and follow-up data. Differences between the composite indices in the two study arms were not statistically significant, although a post hoc analysis suggested the COVID-19 pandemic was a key factor in these results. Qualitative finding suggested that participants and stakeholders generally viewed BETTER WISE positively and emphasized the effects of the pandemic.

**Conclusions and implications for cancer survivors:**

BETTER WISE shows promise for providing an evidence-based, patient-centred, comprehensive approach to prevention, screening and cancer surveillance for cancer survivors in the primary care setting.

**Trial registration:**

ISRCTN21333761. Registered on December 19, 2016, http://www.isrctn.com/ISRCTN21333761.

**Supplementary Information:**

The online version contains supplementary material available at 10.1007/s00520-023-07883-4.

## Introduction

Cancer is a highly prevalent condition worldwide, with the global cancer burden expected to be 28.4 million cases in 2040 [1]. The most common cancers are breast, lung and colorectal for females and lung, prostate and colorectal for males, with these four accounting for approximately 40% of all cancers [1, 2]. Thanks to organized screening programs and continuous improvements in diagnostic tools and treatment, more people are surviving their cancers. As the number of cancer survivors increase, it is crucial that healthcare systems are equipped to address their ongoing surveillance, screening and prevention needs. Cancer survivors are not only at risk of recurrence of their disease, but also remain at risk for other cancers and for other chronic diseases and may require ongoing management of symptoms related to previous treatment.

Cancer screening and chronic disease management are core to the work of primary care, and high-quality guidelines exist for breast, prostate and colorectal cancer survivorship that can be implemented in primary care [3–5]. However, there is a known evidence-to-practice gap in primary care for these patients. For example, in their population-based study, Grunfeld et al. found that 65% of breast cancer survivors were never screened for colorectal cancer and 50% of colorectal cancer survivors were never screened for breast cancer [6]. McBride et al. found considerable variation between Canadian provinces for guideline-based follow-up care, chronic disease management and preventive care for breast cancer survivors in primary care [7]. Evidence-based approaches are needed in the primary care context to support the implementation of high-quality care for cancer survivors.

The BETTER (Building on Existing Tools to Improve Chronic Disease Prevention and Screening in Primary Care) intervention is an evidence-based approach to prevention and screening for cancers and chronic diseases in primary care, proven effective in randomized trials and implementation studies [8–10]. The approach centres around a non-physician health professional in the primary care setting who is trained as a Prevention Practitioner (PP), and who holds focussed prevention visits with patients. Guided by an algorithm that harmonizes high-quality evidence on screening and prevention, the PP develops a prevention prescription for the patient, and supports the patient to set S.M.A.R.T (specific, measurable, attainable, realistic, time-based) health goals through a shared decision-making process [11]. In the original BETTER trial, patients who received the BETTER intervention met 55.6% of screening and prevention actions for which they were eligible at 6 months versus 23.1% for patients in the control group [8].

We subsequently developed BETTER WISE (Building on Existing Tools to Improve Cancer and Chronic Disease Prevention and Screening in Primary Care for Wellness of Cancer Survivors and Patients), an adaptation of BETTER that added comprehensive follow-up for breast, prostate and colorectal cancer survivors to the core elements of the approach. We evaluated BETTER WISE in three Canadian provinces (Alberta, Ontario, and Newfoundland & Labrador) through both a pragmatic cluster randomized control trial and a qualitative evaluation [12]. The BETTER WISE study population included both those with no personal history of cancer and those who were breast, prostate or colorectal cancer survivors. For the latter group, PPs were guided by both a pre-existing algorithm focussed on prevention and screening for cancers and chronic diseases [8] that was updated for BETTER WISE, as well as a newly created cancer surveillance algorithm (see Appendix A). In the current paper, we describe the process of harmonizing cancer survivorship guidelines to create the new BETTER WISE cancer surveillance algorithm and describe both the quantitative and qualitative findings for BETTER WISE participants who were breast, prostate or colorectal cancer survivors. We also describe the results in the context of the coronavirus disease of 2019 (COVID-19) pandemic. The results for the broader BETTER WISE participants are presented elsewhere.

## Methods

### Harmonization of survivorship guidelines for breast, prostate and colorectal cancer survivorship

An evidence review group, consisting of seven researchers and clinicians with primary care and cancer expertise, worked with the Centre for Effective Practice (CEP), a non-profit research consulting group based out of the University of Toronto, to develop a literature search strategy and identify relevant guidelines from web-based repositories and provincial, national and international cancer organizations. Among those identified, we selected guidelines that focussed on adult cancer survivors and were published in English (2009–2016) with a rigorous evidence base, as assessed using select items of the AGREE II (Appraisal of Guidelines for Research and Evaluation) Instrument [13]. More weight was given to the most up-to-date guidelines and those developed by Canadian and American organizations. In order to ensure congruence of the final harmonized algorithm with existing jurisdictional policies and practices, we also considered guidelines from each of the three participating provinces. We excluded guidelines if they were published in a language other than English, did not focus on cancer survivorship or failed to meet the quality criteria. Valued for their local context, guidelines from any of the three provinces were generally not excluded. An expert reviewer then appraised selected guidelines using the full AGREE II Instrument to evaluate quality.

Once identified and assessed, the cancer survivorship guidelines deemed to be of high quality by the AGREE II Instrument were harmonized by a clinical working group (CWG) consisting of 21 members: patients, clinical experts, researchers and health administrators. Members of the CWG independently assigned a vote of yes, no or maybe to specific recommendations (e.g. annual surveillance mammogram for women with a history of breast cancer) from the selected guidelines. Recommendations for which a consensus decision of “no” was reached were excluded from the algorithm. Those for which there were a range of responses were discussed until a consensus of “yes” or “no” was reached. Recommendations for which a consensus decision of “yes” was reached were harmonized by the CWG so that the lowest common requirement (e.g. frequency of testing) was included in the algorithm unless there was evidence to support the higher frequency interval. Where specific frequency recommendations were available for local jurisdictions, these were included in the BETTER WISE cancer surveillance algorithm.

The search identified 26 survivorship guidelines (4 general, 6 breast, 6 prostate and 10 colorectal), of which 9 were rated high quality. These 9 guidelines contained 94 specific recommendations for review [3–5, 14–19]. Based on the review, 15 recommendations were selected for inclusion into a clear and concise BETTER WISE cancer surveillance algorithm tailored for use in primary care (see Appendix A). The algorithm included recommendations related to surveillance testing, bone health, the use of survivorship care plans, managing mental health symptoms, and improving confidence in managing symptoms and long-term effects.

### Cluster randomized trial

Details of the study design have been previously published [12]. We conducted the cluster randomized trial among the patients of 59 family physicians in 13 primary care practices across three Canadian provinces, with each primary care practice having one individual assuming the role of PP. Cluster randomization of patients to intervention versus wait-list control was done at the level of the family physician. As with previous BETTER studies, patients had to be 40–65 years of age as most prevention and screening activities are relevant to this age group [8–10]. Our target was 20 patients per physician, five of whom were expected to be cancer survivors. Cancer survivors were excluded if they were palliative or receiving active treatment, but patients receiving long-term preventive treatment (e.g. aromatase inhibitors) were permitted. Patients were also excluded if they were unable to give informed consent or if we could not access medical records for the previous 3 years. Patients were invited to participate in BETTER WISE by standardized invitation letter. Patient recruitment occurred from January 2018 to August 2019.

Consenting patients who were randomized to the intervention were asked to complete a detailed health survey prior to the visit, which queried information on demographics, lifestyle factors, pre-existing health conditions, quality of life using the EQ-5D instrument [20], mood using the Patient Health Questionnaire-2 (PHQ-2) and Generalized Anxiety Disorder 2-item (GAD-2) scores [21], and other detailed medical history. They then attended an approximately 1-h individual prevention visit with the PP, who had reviewed both their survey results and their medical record. The visit resulted in personalized prevention and cancer surveillance prescriptions that were also shared with the family physician (see Appendices B and C). Patients in the intervention arm completed health surveys and had prevention visits at 6-month intervals up to 24 months after the initial visit. Patients randomized to wait-list control completed the survey after consent and again at 12 months, when their first prevention visit was expected. Prevention visits were conducted in person initially, but a telephone option was added due to the COVID-19 pandemic.

Outcomes were at the individual patient level. Consistent with previous BETTER trials, the primary outcome for this trial was a prevention and screening composite index: the total number of actions met at 12 months divided by the total number of actions for which the patient was eligible to receive at baseline [8]. Data for the composite index came from both survey results and medical record review. Actions included in the composite index were based on the evidence-based recommendations and BETTER WISE algorithm. For cancer survivors, we also created a similarly structured cancer surveillance composite index (see Appendices D and E for details regarding eligibility for, and achievement of, each item in both composite indices).

### Analysis

We used a modified intention to treat (ITT) principle to estimate overall effectiveness of the PP intervention. Both simple means, as well as generalized estimating equation models, with compound symmetric working correlation structure, were used to estimate the difference in accomplishment of our composite outcome between groups randomized to the BETTER WISE intervention arm versus the wait-list control arm. Participants who were missing 12 months follow-up information used for estimation of the composite outcome measure were excluded from our primary analysis.

### Qualitative evaluation

Methods for the qualitative evaluation have been previously published [11, 12]. Briefly, we held interviews with key informants and focus groups with family physicians and their staff including all PPs involved in BETTER WISE across the three provinces to understand perceived impact, barriers and enablers of the approach. All focus group and interview participants provided written informed consent. We conducted focus groups in person at each of the participating primary care clinics at the beginning of the study and then, because of the interruptions of the COVID-19 pandemic, changed to virtual focus groups using Zoom® or telephone at mid-point and at the end of the study. All one-on-one key informant interviews were conducted online over Zoom® or telephone. All focus groups and key informant interviews were audio recorded, transcribed, proofread and edited.

Patients were invited after their prevention visit to provide anonymous feedback using a short feedback form that asked them about their experience of the BETTER WISE visit. Patients received an information letter along with the feedback form, which informed them that by completing the feedback form and submitting it to the team they were providing implied consent to participate in the qualitative component of the project. Patient responses were collected in REDCap®, an electronic data capture tool hosted and supported by the Women and Children’s Health Research Institute at the University of Alberta. We conducted a thematic analysis by filtering out all the data related to cancer and cancer survivorship and identifying main themes through several rounds of coding and discussing the data and emerging themes as a team until consensus was reached.

### Ethics approval

Ethical and operational approval was obtained from the Health Research Ethics Board at the University of Alberta (Pro00067811 and Pro00069064), the Health Research Ethics Board of Newfoundland & Labrador (#2017.027, 2017.027B, 2017.027C, 2017.027D and 2017.284), the Markham-Stouffville Hospital Research Ethics Board (no file number assigned) and the Research Ethics Board at St. Michael’s Hospital (#17-050 and #17-248). All analyses were performed in accordance with the relevant guidelines and regulations.

## Results

### Cluster randomized trial

**Fig. 1:**
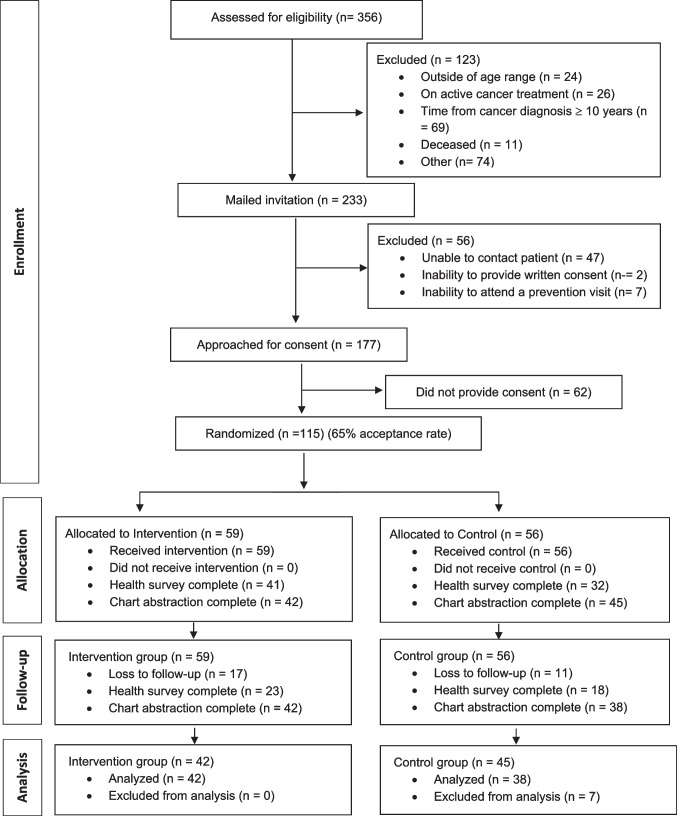
BETTER WISE cancer survivor CONSORT flow diagram

There were 115 cancer survivors eligible, consented and randomized in the BETTER WISE trial. Baseline and 12-month follow-up data were available for 80 patients (42 in the intervention group and 38 in the control group) (see Fig. 1 for CONSORT diagram). Of those 80, there were 47 breast cancer survivors, 17 prostate cancer survivors and 17 colorectal cancer survivors (Table 1). The majority (68.8%) were women, and the mean age was 57.8 years. Approximately 90% of respondents were non-smokers and only seven people reported consuming seven alcoholic drinks or more per week. Over one-third of cancer survivor respondents met the definition for obesity and only 12.5% reported engaging in at least 150 min of physical activity per week. Over one-third reported not being employed, 13.6% reported not having prescription coverage and 25.5% reported a household income of $60,000 CAD per year or less. On the EQ-5D visual analogue scale, survivors reported a mean self-rated health on the day of baseline survey completion of 74.1 (out of 100). Health-related quality of life was generally high, with a mean EQ-5D index score of 0.82 (a score of 1.0 indicates full health). Based on the PHQ-2, 11.8% of survivors reported a possible case of depression requiring diagnostic evaluation and based on the GAD-2, 11.8% reported a possible case of generalized anxiety disorder requiring diagnostic evaluation.

**Table 1:** Baseline demographic characteristics of 80 cancer survivors within BETTER WISE intervention arm and control wait-list arm. Percentage calculations exclude missing from denominator

Descriptive variables at baseline	All *N*=80	Intervention *N*=42	Control *N*=38
Breast cancer (*n*, %)			
1. No	8 (14.5)	3 (10.3)	5 (19.2)
2. Yes	47 (85.5)	26 (89.7)	21 (80.8)
3. Missing (i.e. males)	25	13	12
Prostate cancer (*n*, %)			
1. No	8 (32.0)	5 (38.5)	3 (25.0)
2. Yes	17 (68.0)	8 (61.5)	9 (75.0)
3. Missing (i.e. females)	55	29	26
Colorectal cancer (*n*, %)			
1. No	63 (78.8)	33 (78.6)	30 (78.9)
2. Yes	17 (21.2)	9 (21.4)	8 (21.1)
3. Missing	0	0	0
Sex (*n*, %)			
1. Female	55 (68.8)	29 (69.0)	26 (68.4)
2. Male	25 (31.2)	13 (31.0)	12 (31.6)
3. Missing	0	0	0
Age (mean ± SD)	57.84 (5.64)	58.24 (5.43)	57.40 (5.90)
Current smoker (*n*, %)			
1. No	61 (89.7)	35 (85.4)	26 (96.3)
2. Yes	7 (10.3)	6 (14.6)	1 (3.7)
3. Missing	12	1	11
Current alcohol consumption			
1. 0/week	7 (14.0)	4 (13.8)	3 (14.3)
2. 1–7/week	36 (72.0)	20 (69.0)	16 (76.2)
3. 7–14/week	4 (8.0)	2 (6.9)	2 (9.5)
4. >=14/week	3 (6.0)	3 (10.3)	0 (0.0)
5. Missing	30	13	17
Physically active (*n*, %)			
1. ≥ 150 min/week	8 (12.5)	3 (7.9)	5 (19.2)
2. < 150 min/week	56 (87.5)	35 (92.1)	21 (80.8)
3. Missing	16	4	12
Met definition for obesity, i.e. body mass index of 30 or greater (*n*, %)			
1. Yes	27 (35.5)	14 (33.3)	13 (38.2)
2. No	49 (64.5)	28 (66.7)	21 (61.8)
3. Missing	4	0	4
Canadian citizen by birth (*n*, %)			
1. Yes	53 (79.1)	31 (77.5)	22 (81.5)
2. No	14 (20.9)	9 (22.5)	5 (18.5)
3. Missing data	13	2	11
Race (*n*, %):			
1. White	46 (73.0)	27 (73.0)	19 (73.1)
2. Other	17 (27.0)	10 (27.0)	7 (26.9)
3. Missing data	17	5	12
Highest education level obtained (*n*, %):			
1. Some university or college education	52 (77.6)	33 (82.5)	19 (70.4)
2. Other	15 (22.4)	7 (17.5)	8 (29.6)
3. Missing data	13	2	11
Current employment (*n*, %):			
1. Full/part-time employed	41 (61.2)	25 (62.5)	16 (59.3)
2. Other	26 (38.8)	15 (37.5)	11 (40.7)
3. Missing data	13	2	11
Marital status:			
1. Married/common-law	57 (85.1)	34 (85.0)	23 (85.2)
2. Other	10 (14.9)	6 (15.0)	4 (14.8)
3. Missing data	13	2	11
Last year’s household income (*n*, %):			
1. <$60,000	13 (25.5)	8 (26.7)	5 (23.8)
2. 60,000–99,999	14 (27.5)	9 (30.0)	5 (23.8)
3. 100,000–149,999	14 (27.5)	9 (30.0)	5 (23.8)
4. >=150,000	10 (19.5)	4 (13.3)	6 (28.6)
5. Missing data	29	12	17
Have prescription medication insurance coverage (*n*, %)			
1. Yes	57 (86.4)	34 (87.2)	23 (85.2)
2. No	9 (13.6)	5 (12.8)	4 (14.8)
3. Missing data	14	3	11
Worry about losing place to live (*n*, %)			
1. Always	0 (0.0)	0 (0.0)	0 (0.0)
2. Very often	2 (3.1)	2 (5.4)	0 (0.0)
3. Sometimes	6 (9.4)	5 (13.5)	1 (3.7)
4. Rarely	56 (87.5)	30 (81.1)	26 (96.3)
5. Missing data	16	5	11
Food security status (*n*, %)			
1. Food secure	66 (98.5)	40 (100.0)	26 (96.3)
2. Not food secure	1 (1.5)	0 (0.0)	1 (3.7)
3. Missing data	13	2	11
EQ-5D visual analogue scale (mean ± SD)	74.06 (15.17)	73.88 (12.67)	74.33 (18.51)
EQ-5D quality of life index ^a^ (mean ± SD)	0.82 (0.16)	0.83 (0.13)	0.82 (0.20)
PHQ-2 – Depression score ^b^ (mean ± SD)	0.79 (1.44)	0.85 (1.39)	0.70 (1.54)
PHQ-2 Positive screen (n, %)			
1. No	60 (88.2)	36 (87.8)	24 (88.9)
2. Yes	8 (11.8)	5 (12.2)	3 (11.1)
3. Missing data	12	1	11
GAD-2 – Anxiety score ^c^ (mean ± SD)	1.01 (1.52)	0.98 (1.41)	1.07 (1.71)
GAD-2 Positive screen (*n*, %)			
1. No	60 (88.2)	36 (87.8)	24 (88.9)
2. Yes	8 (11.8)	5 (12.2)	3 (11.1)
3. Missing data	12	1	11

^a^EQ-5D summary index value is derived by applying a formula to values from each dimension of health status (mobility, self-care, usual activity, pain/discomfort and anxiety/depression). Scores range from 0 (as state as bad as being dead) to 1 (full health)

^b^PHQ-2: Patient Health Questionnaire 2 item. Score for the 2 item screen ranges from 0 to 6; score of 3 or more indicates a possible case of depression which requires further diagnostic evaluation

^c^GAD-2: Generalized Anxiety Disorder-2 item. Score for 2 item screen ranges from 0 to 6; score of 3 or more indicates a possible case of generalized anxiety disorder which requires further diagnostic evaluation

Results for individual items in both composite indices are shown in Table 2. There was heterogeneity in eligibility for individual items, with the highest eligibility (*n*=63) for nutrition/diet referral and improvement in healthy diet score, and the lowest for surveillance CT scan (1 person) and surveillance prostate specific antigen (no one eligible). A notable difference between the two groups was seen for having a documented care plan in the chart at 12 months (53.3% in intervention group, 10.0% in control group).

**Table 2:** Met actions at 12 months/eligible actions at baseline for cancer and chronic disease prevention and screening actions and for cancer surveillance actions in cancer survivors

	Action	All	Intervention	Control
Section A	*Actions for prevention and screening*	*n* met at 12 months/*n* eligible at baseline (%)	*n* met at 12 months/*n* eligible at baseline (%)	*n* met at 12 months/*n* eligible at baseline (%)
	Screening			
1	Fasting blood sugar or hemoglobin A1c screening	14/38 (36.8%)	5/18 (27.8%)	9/20 (45.0%)
2	Fasting blood sugar or hemoglobin A1c monitoring	2/2 (100.0%)	1/1 (100.0%)	1/1 (100.0%)
3	Blood pressure screening	11/15 (73.3%)	3/3 (100.0%)	8/12 (66.7%)
4	Blood pressure monitoring	13/13 (100.0%)	8/8 (100.0%)	5/5 (100.0%)
5	Breast cancer screening (women only; *N* = 55)	2/3 (66.7%)	0/0 (0.0%)	2/3 (66.7%)
6	Colorectal cancer screening	12/19 (63.2%)	6/8 (75.0%)	6/11 (54.5%)
7	Cervical cancer screening (women only; *N* = 55)	9/18 (50.0%)	3/6 (50.0%)	6/12 (50.0%)
8	Cardiovascular risk assessment	3/25 (12.0%)	3/11 (27.3%)	0/14 (0/0%)
9	ACE or ARB optimization referral	0/2 (0.0%)	0/2 (0.0%)	0/0 (0.0%)
10	BMI screening	13/32 (40.6%)	6/14 (42.9%)	7/18 (38.9%)
11	Waist circumference measurement	0/26 (0.0%)	0/14 (0.0%)	0/12 (0.0%)
	Treatment			
12	Cholesterol treatment	3/10 (30.0%)	3/5 (60.0%)	0/5 (0.0%)
13	Weight control referral	4/27 (14.8%)	2/14 (14.3%)	2/13 (15.4%)
14	Smoking cessation referral	2/7 (28.6%)	2/6 (33.3%)	0/1 (0.0%)
15	Alcohol cessation referral	5/44 (11.4%)	2/25 (8.0%)	3/19 (15.8%)
16	Physical activity referral	7/56 (12.5%)	5/35 (14.3%)	2/21 (9.5%)
17	Nutrition/diet referral	6/63 (9.5%)	3/38 (7.9%)	3/25 (12.0%)
	Risk modification			
18	Hypertension control	9/20 (45.0%)	7/13 (53.8%)	2/7 (28.6%)
19	Depression score improve	2/8 (25.0%)	2/5 (40.0%)	0/3 (0.0%)
20	At-risk alcohol improvement	5/20 (25.0%)	3/10 (30.0%)	2/10 (20.0%)
21	Low physical activity improve	8/56 (14.3%)	8/35 (22.9%)	0/21 (0.0%)
22	Overweight improvement	29/54 (53.7%)	19/30 (63.3%)	10/24 (41.7%)
23	Smoking cessation	0/7 (0.0%)	0/6 (0.0%)	0/1 (0.0%)
24	Healthy diet score improvement	20/63 (31.7%)	15/38 (39.5%)	5/25 (20.0%)
Section B	***Actions for cancer surveillance***	(*n* met at 12 months/*n* eligible at baseline) %	(*n* met at 12 months/*n* eligible at baseline) %	(*n* met at 12 months/*n* eligible at baseline) %
1	Surveillance prostate specific antigen (PSA)	0/0 (0.0%)	0/0 (0.0%)	0/0 (0.0%)
2	Surveillance colonoscopy	3/6 (50.0%)	2/4 (50.0%)	1/2 (50.0%)
3	Surveillance carcinoembryonic antigen (CEA)	0/2 (0.0%)	0/1 (0.0%)	0/1 (0.0%)
4	Surveillance CT scan	1/1 (100.0%)	1/1 (100.0%)	0/0 (0.0%)
5	Breast cancer surveillance	17/19 (89.5%)	5/7 (71.4%)	12/12 (100.0%)
6	Bone density screen	1/2 (50.0%)	0/0 (0.0%)	1/2 (50.0%)
7	Follow-up care plan	9/25 (36.0%)	8/15 (53.3%)	1/10 (10.0%)
8	Distress score	20/56 (35.7%)	12/35 (34.3%)	8/21 (38.1%)
9	Long-term effects and symptom management confidence improve	5/18 (27.8%)	1/11 (9.1%)	4/7 (57.1%)

Differences between the composite indices in the two study arms were not statistically significant. For the general prevention and screening index, the intervention group was eligible for an average of 8.2 items at baseline, while the control group was eligible for an average of 7.5 items at baseline. At 12 months, the mean composite index was 28.9% for the intervention group vs. 27.1% for the control group, *p*=0.84. For the cancer surveillance composite index, the intervention group was eligible for an average of 1.8 items at baseline, while the control group was eligible for an average of 1.5 eligible items. At 12 months, the mean composite index was 35.2% for the intervention group vs. 52.7% for the control group, *p*=0.07.

As our study took place during the COVID-19 pandemic when many screening and preventive aspects of the health system were put on pause, we conducted a post hoc analysis, where we examined composite index results for the 7 cancer survivors in the intervention group and 10 cancer survivors in the control group whose results were measured prior to the pandemic (before February 2020). For this analysis, we used per protocol estimates of the PP intervention effect. Prior to the pandemic, the results for the general prevention and screening composite index were 34.8% vs. 8.0% (intervention vs. control). For the cancer surveillance composite index, the results were 41.7% vs. 16.7% (intervention vs. control).

## Qualitative results

For the BETTER WISE study, 132 primary care staff (PPs, family physicians, allied health professionals, clinic staff and a research assistant) participated in 17 focus groups and 48 key informant interviews from the 13 participating primary care settings in the three provinces. Qualitative data were collected at three points (baseline, follow-up and final interview). Eighty-five patient feedback forms were received from cancer survivors.

In our thematic analysis of feedback forms from cancer survivors and interviews and focus groups with stakeholders, family physicians and PP participants, there were several key themes that emerged: the benefit of the PP role for cancer survivors, patient engagement and attitudes toward goal setting, and the impact of the COVID-19 pandemic.

### Benefit of Prevention Practitioner (PP) role for cancer survivors

Patients, PPs and family physicians alike expressed their appreciation for the addition of the PP role. Physicians believed that patients were receiving better care as a result. Some PPs viewed their role as that of a navigator or connector between patients and their family physicians and/or oncologists. They followed up with other health practitioners to answer patients’ questions or to fill in the gaps where patients had forgotten their cancer survivorship/surveillance care plans.

I was able to find out the name of their… oncologist and find out exactly what that care plan was supposed to be… it was more like a navigator position where I was between all of these resources and this person and then I was able to make a connection between the oncologist and the doctor or the oncologist and the patient. And so, I would say that the most valuable piece that came out of it was just revisiting that and making the connections. [PP, KI033, AB]

On the receiving end, many patients expressed their appreciation that PPs were willing to connect with their oncologists and able to answer their questions or help with goal setting.

[The PP] was very willing to work/speak to my oncologist and problem solve for me. I didn't expect that and really appreciated her help. [Patient, female, AB]

For many patients, visits with PPs were opportunities to speak openly in a judgment-free environment with someone who provided validation for their concerns and motivation to make them feel more in charge of their health.

[The prevention visit] reminded me of things I need to remember - dates, timing and helped make me feel remembered and valued… and that my life is still important! [Patient, female, AB]

Patients appreciated the longer, unrushed nature of their visits with PPs. They felt that their PPs were well-prepared, and that their visits were thorough.

Lots of time to discuss things in detail [in the prevention visit]. [It was] not rushed… The visit was very thorough. [Patient, female, AB]

Overall, cancer survivor patients greatly appreciated having one on one visits with PPs to make sure they were meeting their screening requirements.

I love that [the PP] went through all the dates for my next cancer follow up screenings. [Patient, female, ON]

Consequently, PPs felt that the work was valuable and rewarding, especially when their visits resulted in positive outcomes for patients.

However, many PPs commented that the visits for cancer survivor patients required more preparation time or resulted in longer visits than for BETTER WISE participants who were not cancer survivors, as there was more information to cover and particularly if patients chose to set goals. Preparation for cancer survivors could be more time-intensive if PPs were checking reports from oncologists or searching for care plans to find more information for their patients.

[Prevention visits for] the cancer survivors were definitely a lot more work in terms of time… it took time to find their care plan on their chart … there was a lot more digging to figure out and to confirm when exactly the screening was supposed to be… So, I ended up making a few phone calls or just double-checking what exactly the [screening] interval was supposed to be for that person… [PP, KI033, AB]

One PP felt that more skill was needed for the visits with cancer survivor patients and that they had less capacity to answer questions. However, as they became more familiar with the material, over time their comfort level increased.

Well, I had less practice with the cancer survivors, so I really noticed – I noticed my skill level increasing over time as I practiced. [PP, KI033, AB]

### Patient engagement and attitude towards goal setting

The PPs noted that there was great variation in the attitudes of cancer survivor participants. They believed that most patients were interested and engaged in their prevention visits.

Many patients had very positive responses to the study, and some were motivated to participate in it so that others would benefit in the future. These patients were receptive, invested and enjoyed the program.

Being a cancer survivor, I wish I could help and support others dealing with this dreadful disease. Having someone who has been through the experience would be so beneficial. [Patient, female, AB]

However, PPs noted that other patients were less engaged or more reluctant to engage. Some patients were not interested in setting specific goals. Others already had health goals that they were actively working on. There were also patients who regularly saw their oncologist and did not feel that they needed additional support. However, some patients who were at first reluctant to participate in goalsetting became more engaged over time.

[Prevention visits] might be more useful for folks who had not already reviewed issues with doctors and dietitians [Patient, male, ON]

[T]here were patients that initially didn’t seem motivated… like, the first visit, they seemed a bit guarded and they didn’t want to make any goals. But then the second visit they opened up a bit and then they were ready to make goals [PP, KI046, AB]

Some PPs noted that where patients were in their cancer survivorship journey also varied as did their likelihood of being on track with cancer surveillance:

And a lot of the cancer survivors to our site… a lot of them are… [past] that… five-year mark since they've been diagnosed. So, a lot of them have been discharged from cancer clinics and just ongoing kind of regular care with their physician. So, for the most part, they were able to stay on track with cancer screening and bloodwork and whatever else that they might need to do as part of their cancer screening [PP, KI036, ON]

Like the cancer surveillance itself it was almost like it wasn’t there. The only thing that I could see there was the mammogram… the other stuff, like the bone density, one lady did that every five years, but it was only because I brought it up…. So, like it wasn’t obvious… there was no planning in her chart that I could see and they were both around ten years… And the long-term effects, like one lady actually does have a lot of pain, residual bone pain… ever since she’s been diagnosed ten years ago. And you know her treatment plan was outlined for that. You know but the rest of it, it wasn’t real obvious... [Prevention Practitioner, KI039, NL]

Patient disengagement, especially for patients earlier on in their cancer survivorship journey, may also have been explained by appointment fatigue. One patient mentioned their frustration with multiple appointments and the lack of communication between their health care providers that resulted in always having to re-explain their situation to practitioners.

[I] have multiple specialists and a FP [family physician] … people don't always seem to know what the other is doing … lots of appointments and this is another one! ... tired of always talking and explaining my scenario. [Patient, female, AB]

### The impact of the COVID-19 pandemic on cancer screening and surveillance

The COVID-19 pandemic was declared in March 2020 and the study, along with cancer screening and surveillance overall, was greatly affected. Details have been documented elsewhere [22]. For physicians and PPs, the pandemic resulted in increased COVID-related clinical responsibilities, which took priority over preventive care. Across the country, various cancer screening programs were suspended or delayed. Along with delayed testing and screening backlogs, some patients were hesitant to come into clinics or chose to postpone their screening.

You know, I’ve offered a few of them, like, just to come in for a blood pressure reading and they are hesitant and just … a few people haven’t wanted to do their appointment at all and wanted to wait a while. So, it’s just been at the back of the queue. [PP, KI031, ON]

However, this may have been more of an issue at the start of the pandemic before a vaccine was available and when fewer treatment options existed. Physicians and PPs eventually noted their attempts to catch up on screening and clear the backlog was received positively by patients.

Yeah, most of my patients who I've asked them to come in have been coming in. I haven't had too many where they're like, ‘oh, I don't want to come in because of the pandemic’. I think that happened a lot more in the first six months I want to say. But I would say that since the summer… I have started essentially calling my patients that were due for screening. I still manage a lot on the phone, but those who need their mammograms and their PAP smears and their FIT tests and stuff, we're ordering them [PP, KI048, AB]

One PP expressed concern about the isolation of immunocompromised cancer survivor patients during the COVID-19 lockdown.

[Prevention visits] were very sad because that's exactly the population that was extremely high risk, because [two cancer survivor patients] both had other issues after the cancer, [being] immunocompromised. Just like so isolated, so missing the freedom to go somewhere without being afraid, like they don't even go grocery shopping. I just liked to talk to them and try to, I don't know, give them a new voice to listen to [PP, KI034, ON]

At the time of final data collection, some clinics had not yet returned to outreach screening and were still screening opportunistically. Some physicians and PPs commented that they expected some fallout due to paused or delayed screening during the pandemic and expected to see a higher number of pathologies over the next few years.

I think down the road, yes, the screening is, there’s a two-year gap in how much screening people have done. I’m sure there’s going to be a bit of a fall out from that over the next two years when the screening gets done. I would think the pick-up things might be a little bit higher, but I think for us, what we’re seeing in healthcare right now is we’re seeing more acute presentations of illness like in emergency here right now, emerg[ency] is kind of overwhelmed [Physician, KI040, NL]

As a result of pandemic backlogs for screening and routine health visits, physicians believed that PPs would be especially useful at this time to reach out to high-risk patients, patients with overdue screening, and to improve screening practices at the clinic overall.

I think [having a prevention practitioner] would be an interesting or a very useful addition potentially to primary care practice if we could find a way to make that work, especially right now during COVID. I think, well we're trying to do, what we can in terms of catching people with screening or preventative health as we see them for issues, like we haven't really been doing routine physicals, which is normally when all of this preventative health and screening would have been done. So especially now when we're still limited in terms of office visits and there's still all these other kind of more acute issues that are kind of taking up more office time, I think it would be useful to have like this Prevention Practitioner if it was available to reach out to certain high risk group patients to ensure that they are up to date with their screening. [Physician, FG005, ON]

## Discussion

In the primary care–based BETTER WISE intervention, PPs held visits focussed on prevention and screening, and including a cancer surveillance component for participants who were breast, colorectal and/or prostate cancer survivors. Visits were guided by an evidence-based general prevention and screening algorithm as well as by a cancer surveillance algorithm for cancer survivors that was based on review and harmonization of high-quality cancer survivorship guidelines. We evaluated BETTER WISE in a cluster randomized trial using two composite indices (a general composite index applicable to all BETTER WISE participants and a cancer survivor composite index only applicable to those patients who are cancer survivors), as well as through qualitative evaluation. Among our 80 cancer survivors for whom we had 12-month follow-up data (42 in intervention group, 38 in control group), differences between the two study arms were not statistically significant at 12 months; however, promising results prior to the COVID-19 pandemic suggested that pandemic-related system shutdowns likely played a substantial role in this finding. Only 17 cancer survivor participants reached the 12-month outcome prior to the pandemic being declared, which affected the power of the study to detect any between-group differences. Qualitative analysis showed that patients, PPs and physicians generally viewed BETTER WISE positively and appreciated the comprehensive, patient-centred and evidence-based approach. PPs noted that extensive time, training and experience were needed for their role to be successful to support care for cancer survivors. Some cancer survivors were not as engaged in BETTER WISE, particularly if it was earlier in their cancer journey or if they already felt well supported by their oncology team, but BETTER WISE created an opportunity to ensure cancer survivors, particularly those without a documented care plan, were receiving evidence-based cancer surveillance.

Qualitative findings reinforced that the COVID-19 pandemic impacted BETTER WISE; the pandemic led to system-wide pauses and delays in prevention and screening, and to social isolation, which may have taken an even higher toll on cancer survivors. The PP visits may have been a helpful touchpoint during times of social isolation. It has been documented that cancellation and postponement of cancer testing due to the pandemic resulted in increased fear and anxiety for many patients across Canada [23]. Further studies on the impact of the pandemic on this population, and interventions to address it, are required as cancer survivors navigate new means of healthcare delivery and challenges related to uncertainty and social isolation [24].

Family physicians play a critical role in cancer survivorship care, and studies have shown that preventive services for cancer survivors are more likely to be provided when the survivor is followed by both an oncologist and a family physicians, rather than just one or the other [25] However, many obstacles exist for family physicians, such as inadequate knowledge about the long-term needs of cancer survivors, challenges keeping up with a highly specialized knowledge area, and lack of trust in their abilities by oncologists and patients [25]. Survivorship care plans have been highlighted as a tool that may increase confidence among family physicians, but it has also been highlighted that these care plans require careful consideration of how they are implemented to maximize utility [26–28]. Of note, 25 of our 80 cancer survivors did not have a follow-up care plan documented in their medical chart at study onset. A systematic review by Ke et al. found that oncologists sometimes lack confidence in family physicians’ ability to provide follow-up care and surveillance, and recommended that family physicians be trained and empowered to deliver evidence-based survivorship care [29]. Additionally, encouraging healthy behaviours and addressing psychosocial needs of survivors, well within the skillset of family physicians, have been proposed as essential aspects of effective cancer survivorship care [30]. The BETTER WISE intervention may be a powerful way to address the above concerns and achieve the recommendation from Ke et al. Similarly, Alfano et al.’s review of implementation efforts in multiple countries suggested that current cancer follow-up models in many countries fail to meet patients’ needs and recommended, among others, algorithms to triage patients to pathways, methods to assess patient issues to guide care, methods to support patients in self-management and ways to coordinate information exchange between oncologists, family physicians and patients [31]. Again, the BETTER WISE intervention has the potential to achieve these recommendations. Despite this potential, the long-term success and sustainability of BETTER WISE, or any similar intervention for cancer survivors in primary care, will require motivated stakeholders willing to provide permanent investments in resources, funding and personnel [28].

This study had several limitations. First, as discussed, the timeline of our trial included a period where screening and prevention activities were put on pause at a system level, affecting our study outcome in both arms. Anecdotally, we heard from participating clinics that when screening and prevention were resumed, there was more of a focus on catching up those who were overdue, which meant our control group likely did not receive usual care. Second, this sub-analysis was underpowered. We expected a total of 295 cancer survivors but only 115 were randomized, for which only 80 provided 12-month follow-up data and only 17 provided 12-month data prior to the COVID-19 pandemic. Third, some BETTER WISE visits were conducted in person, and some were conducted virtually due to the pandemic. We do not have data to determine if one visit type performed better than the other for cancer survivors. Finally, we limited our study to survivors of breast, prostate and colorectal cancer, and did not include other cancer survivors. However, these cancers are among the most common in Canada and have multiple high-quality guidelines to guide survivorship care.

In April 2020, the editors of the Journal of Cancer Survivorship published a commentary reminding healthcare providers that cancer survivors need tailored, patient-centred care during the trying times of the pandemic and beyond [32]. Despite the null trial findings, our qualitative results and pre-pandemic findings suggest that BETTER WISE shows promise for providing an evidence-based, patient-centred, comprehensive approach to prevention, screening and cancer surveillance for cancer survivors in the primary care setting.

## Data Availability

The datasets generated during and/or analyzed during the BETTER WISE study will not be made publicly available due to planned analyses and publications but are available from the corresponding author on reasonable request. We will not provide full transcriptions of the qualitative data as they may contain quotes and identifiable information that could compromise the identity of participants.

## Supplemental information – appendices

Supplementary File 1 File Name: Lofters - Appendix A - BETTER Cancer Surveillance algorithm. File format: PDF. Title and description: The BETTER Cancer Surveillance Care Map. Supplementary File 2 File Name: Lofters - Appendix B - BETTER Primary Prevention Prescription. File format: PDF. Title and description: The BETTER WISE Primary Prevention Prescription. A summary of the patient’s cancer and chronic disease prevention and screening status. Supplementary File 3. File Name: Lofters - Appendix C - BETTER Cancer Surveillance Prescription. File format: PDF. Title and description: The BETTER WISE Cancer Surveillance Prescription. A summary of the patient’s cancer surveillance status. Supplementary File 4. File Name: Lofters - Appendix D - BETTER WISE Primary Prevention & Screening Composite Index. File format: PDF. Title and description: The BETTER WISE Project Primary Outcome Source Document: Primary Prevention and Screening Composite Index Supplementary File 5 File Name: Lofters - Appendix E - BETTER WISE Cancer surveillance Composite Index. File format: PDF. Title and description: The BETTER WISE Project Primary Outcome Source Document: Cancer Surveillance Composite Index

## Abbreviations

AGREE II: Appraisal of Guidelines for Research and Evaluation Instrument
BETTER: Building on Existing Tools to Improve Chronic Disease Prevention and Screening in Primary Care
BETTER WISE: Building on Existing Tools to Improve Cancer and Chronic Disease Prevention and Screening in Primary Care for Wellness of Cancer Survivors and Patients
CAD: Canadian dollars
CEP: Centre for Effective Practice
COVID-19: coronavirus disease of 2019
CWG: clinical working group
GAD-2: Generalized Anxiety Disorder 2-item
PHQ-2: Patient Health Questionnaire-2
PP: Prevention Practitioner
REDCap: research electronic data capture
S.M.A.R.T: specific, measurable, attainable, realistic, time-based
