# Supplementary material for: Opportunities to improve quality of care for cancer survivors in primary care: findings from the BETTER WISE study
Source: Support Care Cancer. 2023 Jun 30;31(7):430. doi: 10.1007/s00520-023-07883-4 (PMC10313555; doi:10.1007/s00520-023-07883-4)
Supplement: Supplementary File 4. — File Name: Lofters - Appendix D - BETTER WISE Primary Prevention & Screening Composite Index. File format: PDF. Title and description: The BETTER WISE Project Primary Outcome Source Document: Primary Prevention and Screening Composite Index [file 520_2023_7883_MOESM4_ESM.pdf]

Article Title: Opportunities to Improve Quality of Care for Cancer Survivors in Primary Care: Findings from the BETTER WISE Study

Journal Name: Journal of Cancer Survivorship

Author Names: Aisha Lofters, Ielaf Khalil, Melissa Shea-Budgell, Christopher Meaney, Nicolette Sopcak, Carolina Fernandes, Rahim Moineddin, Denise Campbell-Scherer, Kris Aubrey-Bassler, Donna Patricia Manca, Eva Grunfeld.

Corresponding Author: Dr. Aisha Lofters

Corresponding Author Affiliations:

1. Department of Family and Community Medicine, University of Toronto, 500 University Ave, Toronto, Ontario M5G 1V7, Canada
2. Peter Gilgan Centre for Women's Cancers, Women's College Hospital, 76 Grenville St, Toronto, ON M5S 1B2

Corresponding Author Email: aisha.lofters@utoronto.ca

BETTER WISE™

Building on Existing Tools to Improve Cancer and Chronic Disease Prevention and Screening in Primary Care for Wellness of Cancer Survivors and Patients

The BETTER WISE Project

Primary Outcome Source Document:

Cancer and Chronic Disease Prevention and Screening (CCDPS)

Primary Prevention and Screening Composite Index

April 6, 2021

SECTION A: GENERAL INFORMATION 2

SECTION B: CDPS Primary Prevention & Screening Composite Index Table..... 3

SECTION C: INDICATORS AND RANGES..... 9

REFERENCES..... 1

SECTION A: GENERAL INFORMATION

Purpose of this document

This document was prepared from the recommendations made by the Clinical Working Group for items regarding primary prevention and screening for cancer, cardiovascular disease and diabetes (including lifestyle factors). Information on the criteria identified for each item or "criteria for eligibility (E)" AND "criteria for achievement (A)" listed within is to be collected and analyzed for the project's primary analysis. All recommendations were based on 2017 guidelines to improve quality in family practice.

This document contains the **Cancer and Chronic Disease Prevention and Screening (CCDPS) Composite Index Table** which define the referrals/actions patients are deemed eligible (E) to achieve based on assessments at the baseline evaluation and the criteria that must be met in order for a patient to have achieved (A) each referral/action they have been determined to be eligible for. All CCDPS items identified by the Clinical Working Group have been compiled to create the BETTER WISE Main Composite Index. These items include process, referral/treatment, and target/change actions. As there is an association between process measures, referral/treatment actions, and patient outcomes (target/change outcomes), items encompassing these three areas have been included in the Main Composite Index.

- The **BETTER WISE Main CCDPS Composite Index Table (Section B.1),** defines the criteria used to calculate the main outcome CCDPS composite index for the project. The items described in this table allow for a calculation of the proportion change in unachieved CCDPS items for each patient and includes *process, referral/treatment, and target/change actions*. This composite index is calculated at follow-up (12 and 24 months, where applicable). The eligible actions are determined at baseline using the "criteria for eligibility (E)" and at each follow-up evaluation the achievement of the eligible actions is assessed using the "criteria for achievement (A)".
- Where the term Health Professional appears in this document, it refers to Primary Care Practitioner, Nurse, Nurse Practitioner, Prevention Practitioner, Dietician, Nutritionist, Pharmacist, and Clinical Assistant.

- **Indicators and Ranges (Section C)**, which specify the targets/ranges used to determine a patient's eligibility (E) for and achievement (A) of the referrals/actions included in the composite index at each evaluation time point (i.e. baseline, 12 month, and 24 month, where applicable).

This document was created by Dr. Eva Grunfeld, Dr. Donna Manca, Dr. Kris Aubrey-Bassler, Dr. Denise Campbell-Scherer, Dr. Aisha Lofters, Ms. Melissa Shea-Budgell, Dr. Rahim Moineddin, Christopher Meaney, Dr. Paul Kruger, Dr. Nicolette Sopcak, and Ms. Carolina Fernandes from the BETTER WISE Project team.

SECTION B: CDPS PRIMARY PREVENTION & SCREENING COMPOSITE INDEX TABLE

Defines the criteria used to calculate the main composite index for the project, which includes *monitoring and screening actions*. This composite index is calculated at each follow-up evaluation (12 and 24 month, where applicable). The eligible actions are determined at baseline using the "criteria for eligibility (E)" and at each evaluation, the achievement of the eligible actions is assessed using the "criteria for achievement (A)". Items followed by a "*", "**", "***" are process outcome, referral/treatment outcome or target/change outcomes, respectively.

#	Referral/Action	Criteria for Eligibility (E) obtained and set at baseline	Criteria for Achievement (A) at follow-up visits every 6 months	References
1	FBS or HbA1C Screen*	All patients without diabetes with at least one risk factor (see Section C) that have not had a FBS or HbA1C completed in past year	FBS or HbA1C completed	1
		All patients without diabetes without risk factors (see Section C) that have not had a FBS or HbA1C completed in past 3 years	FBS or HbA1C completed	
2	FBS or HbA1C Monitor*	All patients without diabetes with history of impaired FBS (6 - 6.9) or HbA1C (6.0-6.4) that have not had a FBS or HbA1C completed in past 6 months	FBS or HbA1C completed	1
3	BP Screen*	All non CVD patients AND Non-hypertensive and without diabetes that have not had their BP checked in the past 12 months	BP checked	2-4
		All non CVD patients AND Non-hypertensive and patients with diabetes that have not had their BP checked in the past 6 months	BP checked	1,3,5
4	BP Monitor*	All non CVD patients with hypertension that have not had their BP checked in the past 6 months	BP checked	2,3,5
5	Breast Cancer Screen*	All women 50-65 without a personal history of breast cancer AND without elevated risk of breast cancer (see Section C) that have not had a routine mammogram completed in the past 2 years	Mammography complete	6-17
		All women 40-65 without personal history of breast cancer but with elevated risk of breast cancer (see Section C) that have not had a routine mammogram completed in the past year	Mammography complete	
6	CRC Screen*	All patients 50-65 without personal history CRC and without elevated risk of CRC or a known genetic marker (see Section C) that have not completed CRC screening as per the following:<br>o FOBT/FIT done within 2 years OR<br>o Sigmoidoscopy done within 10 years OR	CRC screening completed as per one of the following:<br>• FOBT or FIT;<br>• Sigmoidoscopy; or<br>• Colonoscopy.	18-22

		○ Colonoscopy done within 10 years if normal OR<br>○ Colonoscopy done within 5 years if abnormal		
		All patients 50-65 in Newfoundland & Labrador without a personal history of CRC, but with one 1st degree relative with CRC diagnosis at ≥ 60 years of age that have not completed an FOBT or FIT in the past 2 years	CRC screening completed using FOBT or FIT.	
		All patients 40-65 in Alberta without a personal history of CRC, but with one 1st degree relative with CRC diagnosis at ≥ 60 years of age that have not completed a FIT in the past 2 years	CRC screening completed using FIT	
		All patients 40-65 without a personal history of CRC, with elevated risk of CRC or known genetic marker (see Section C) that have not completed CRC screening as per the following:<br><br>○ Colonoscopy done within 10 years if normal OR<br>○ Colonoscopy done within 5 years if abnormal	CRC screening completed using colonoscopy	
7	Cervical Cancer Screen*	All women without personal history of cervical cancer AND with no abnormalities detected in previous pap tests AND without a hysterectomy with removal of the cervix AND who are not immunocompromised (see Section C) that have not completed routine cervical cancer screening in the past 3 years	Pap test complete	23-26
		All women without personal history of cervical cancer AND with no abnormalities detected in previous pap tests AND without a hysterectomy with removal of the cervix AND who are immunocompromised (see Section C) that have not completed routine cervical cancer screening in the past year	Pap test complete	
8	CVD risk assessment	All men > 40 years old without established CVD or familial hypercholesterolemia whose CVD risk has not been assessed in the past 3 years using QRISK2 or Framingham	CVD risk assessment completed using QRISK2	2,3,27,28
		All women > 50 years old without established CVD or familial hypercholesterolemia whose CVD risk has not been assessed in the past 3 years using QRISK2 or Framingham	CVD risk assessment completed using QRISK2	
		All women 40-50 who are post-menopausal without established CVD or familial hypercholesterolemia whose CVD risk has not been assessed in the past 3 years using QRISK2 or Framingham	CVD risk assessment completed using QRISK2	
		All patients 40-65 with type 2 diabetes without established CVD or familial hypercholesterolemia whose CVD risk has not been assessed in the past 3 years using QRISK2 or	CVD risk assessment completed using QRISK2	

		Framingham		
9	ACE/ARB treatment	All patients 40-65 without established CVD or familial hypercholesterolemia not currently on an ACE or ARB, but there is evidence for prescribing the medication (See Section C).	Referral to primary care provider for ACE/ARB discussion.	1,2,5
10	BMI Screen*	All patients that have not had a BMI measurement in the past 2 years	BMI complete	29-31
11	Waist Circumference*	All patients with BMI 25 to 29.9 that have not had a waist circumference measurement in the past 2 years	Waist Circumference complete	29
12	Cholesterol Treatment**	All men > 40 years old without established CVD or familial hypercholesterolemia whose QRISK2 cardiovascular risk score is ≥ 10%, but < 20% AND not on a statin	• Discussion with primary care provider, internal resource or external resource in progress or complete for cholesterol medication discussion and management.	27
		All women > 50 years old without established CVD or familial hypercholesterolemia whose QRISK2 cardiovascular risk score is ≥ 10%, but < 20% AND not on a statin	• Discussion with primary care provider, internal resource or external resource in progress or complete for cholesterol medication discussion and management.	
		All women 40-65 who are post-menopausal without established CVD or familial hypercholesterolemia whose QRISK2 cardiovascular risk score is ≥ 10%, but < 20% AND not on a statin	• Discussion with primary care provider, internal resource or external resource in progress or complete for cholesterol medication discussion and management.	
		All patients 40-65 with type 2 diabetes without established CVD or familial hypercholesterolemia whose QRISK2 cardiovascular risk score is ≥ 10% AND not on a statin	• Discussion with primary care provider, internal resource or external resource in progress or complete for cholesterol medication discussion and management.	
		All men > 40 years old without established CVD or familial hypercholesterolemia whose QRISK2 cardiovascular risk score is ≥ 20% AND not on a statin	Prescribed cholesterol medication (e.g. statin)	
		All women > 50 years old without established CVD or familial hypercholesterolemia whose QRISK2 cardiovascular risk score is ≥ 20% AND not on a statin	Prescribed cholesterol medication (e.g. statin)	
		All women 40-65 who are post-menopausal without established CVD or familial hypercholesterolemia whose QRISK2 cardiovascular risk score is ≥ 20% AND not on a statin	Prescribed cholesterol medication (e.g. statin)	
		All patients 40-65 with type 2 diabetes without established CVD or familial hypercholesterolemia whose QRISK2 cardiovascular risk score is ≥ 20% AND not on a statin	Prescribed cholesterol medication (e.g. statin)	

13	Referral Weight Control**	Previous BMI ≥ 30	Referral for weight control achieved through:<br>• Referral to primary care provider, internal resource or external resource in progress or complete for nutrition/diet OR<br>• Referral to internal program or external program in progress or complete for any one of Nutrition/diet; Physical activity/exercise; Weight control program OR<br>• Discussion with primary care provider, internal resource or external resource in progress or complete for any one of Nutrition/diet; Physical activity/exercise; Weight control OR<br>• Patient initiated referral for weight control to primary care provider, internal resource/program or external resource/program in progress or complete	31,32
		Previous BMI <30 AND high waist circumference (see Section C)	Referral for weight control achieved through:<br>• Referral to primary care provider, internal resource or external resource in progress or complete for nutrition/diet OR<br>• Referral to internal program or external program in progress or complete for any one of Nutrition/diet; Physical activity/exercise; Weight control program OR<br>• Discussion with primary care provider, internal resource or external resource in progress or complete for any one of Nutrition/diet; Physical activity/exercise; Weight control OR<br>• Patient initiated referral for weight control to primary care provider, internal resource/program or external resource/program in progress or complete	
14	Referral Smoking Cessation**	Person currently smokes	Referral for smoking cessation achieved through:<br>• Newly prescribed smoking cessation medication OR<br>• Referral to primary care provider, internal resource/program or external resource/program for smoking cessation in progress or complete OR<br>• Discussion with primary care provider, internal resource or external resource regarding smoking cessation or smoking cessation medication in progress or complete OR<br>• Patient initiated referral for smoking cessation to primary care provider, internal resource/program or external resource/program (includes cessation aides) in progress or complete	27,33-35

15	Referral Alcohol Discussion**	> 14 drinks per week for Men without a personal history of prostate cancer OR colorectal cancer OR<br>> 0 drinks per week for Men with a personal history of prostate cancer OR colorectal cancer OR<br>Identified as a binge drinker (≥5 drinks on one occasion)	Referral to discuss healthy alcohol consumption, alcohol assessment and advice. Family physician to determine next steps such as:<br>• Newly prescribed alcohol cessation medication OR<br>• Referral to primary care provider, internal resource/program or external resource/program for alcohol cessation in progress or complete, OR<br>• Discussion with primary care provider, internal resource or external resource regarding alcohol cessation in progress or complete OR<br>• Patient initiated referral for alcohol cessation to primary care provider, internal resource/program or external resource/program (includes cessation aids) in progress or complete	27,36,37
		> 7 drinks per week for Women without a personal history of breast cancer OR colorectal cancer OR<br>> 0 drinks per week for Women with a personal history of breast cancer OR colorectal cancer OR<br>Identified as a binge drinker (≥4 drinks on one occasion)	Referral to discuss healthy alcohol consumption, alcohol assessment and advice. Family physician to determine next steps such as:<br>• Newly prescribed alcohol cessation medication OR<br>• Referral to primary care provider, internal resource/program or external resource/program for alcohol cessation in progress or complete, OR<br>• Discussion with primary care provider, internal resource or external resource regarding alcohol cessation in progress or complete OR<br>• Patient initiated referral for alcohol cessation (program, counseling or cessation aid) in progress or complete	
16	Referral Physical Activity**	All patients 40-65 who do <150 minutes of physical activity/week.<br><br>For the purposes of this study, this is defined as a score other than "active" on the General Practice Physical Activity Questionnaire (GPPAQ)	Referral for discussion regarding physical activity achieved through:<br>• Referral to primary care provider, internal resource/program or external resource/program for physical activity/exercise in progress or complete OR Discussion with primary care provider, internal resource or external resource regarding physical activity/exercise in progress or complete OR<br>• Patient initiated referral for physical activity/exercise to primary care provider, internal resource/program or external resource/program in progress or complete	38-42
17	Referral Nutrition/Diet**	All patients 40-65 with an unhealthy diet score.<br><br>For the purposes of this study, this is defined as a score ≥4 on the Starting the Conversation questionnaire.	Referral for discussion regarding nutrition/diet achieved through:<br>• Referral to primary care provider, internal resource/program or external resource/program for nutrition/diet in progress or complete OR	1,42,43

			• Discussion with primary care provider, internal resource or external resource regarding nutrition/diet in progress or complete OR<br>• Patient initiated referral for nutrition/diet to primary care provider, internal resource/program or external resource/program in progress or complete	
18	Hypertension Control***	All patients without CVD and diabetes with hypertension	BP ≤ 140/90	2,5
		All patients without CVD and with diabetes and hypertension	BP ≤130/80	
19	Depression Score Improve*	All patients 40-65 AND who score ≥ 3 points on the Patient Health Questionnaire 2 question screen (PHQ-2).	Improvement in PHQ-2 score (i.e. score <3 points) since previous visit	44-47
20	* Alcohol Improve	> 7 drinks per week for Women without a personal history of breast cancer or colorectal cancer OR<br>> 14 drinks for Men without a personal history of colorectal cancer or prostate cancer	Improvement in alcohol consumption towards suggested targets (i.e. decrease in alcohol consumption) since previous visit	27,36,37
		> 0 drinks per week for Women with a personal history of breast cancer OR colorectal cancer OR<br>> 0 drinks per week for Men with a personal history of prostate cancer OR colorectal cancer		
		≥4 drinks at one time for Women or ≥5 drinks at one time for Men	Improvement in alcohol consumption towards suggested targets (i.e. decrease in binge drinking frequency; Never > Less than monthly > Monthly > Weekly > Daily or almost daily) since previous visit	
21	*Low Physical Activity Improve	All patients 40-65 who do <150 minutes of physical activity/week.<br><br>For the purposes of this study, this is defined as a score other than "active" on the General Practice Physical Activity Questionnaire (GPPAQ)	Improvement in GPPAQ score (Active > Moderately Active > Moderately Inactive > Inactive) since previous visit	38-42,48
22	*Weight Stability	Previous BMI ≥ 25	No increase in weight measured in lbs or kgs including decimal places since previous visit	49,50
23	*Smoking Cessation	Previous documented visit as person currently smoking	Stopped smoking since previous visit	35
24	*Diet Score Improve	All patients 40-65 with an unhealthy diet score.<br><br>For the purposes of this study, this is defined as a score ≥4 on the Starting the Conversation questionnaire.	Improvement in Starting the Conversation diet score (i.e. decrease in diet score) since previous visit	1,42,43,51

SECTION C: INDICATORS AND RANGES

**Domain**	**Target/Range**	**References**
FBS	Normal <6mmol/L; Impaired Fasting Glucose 6 – 6.9 mmol/L; Suspected Diabetes/Diabetes >6.9 mmol/L	1,52,53
HbA1c	Normal < 6%; Impaired Fasting Glucose 6 – 6.4%; Suspected Diabetes/Diabetes ≥ 6.5%	
BP – No hypertension, no CVD	Patients without diabetes target is ≤140/90 (recheck 12 months) and Patients with diabetes target is ≤ 130/80 (recheck 6 and 12 months)	2,41
BP – With hypertension, no CVD	Patients without diabetes target is ≤140/90 (> 140/90 recheck 6 and 12 months) and Patients with diabetes target is ≤ 130/80 (> 130/80 recheck 6 and 12 months)	
Diabetes Screening	If no risk factors, order fasting blood sugar (FBS) or Haemoglobin A1c (HbA1c) every 3 years. If risk factors order FBS or HbA1c every year.	1
Diabetes Risk Factors	If patient meets one or more of the following, patient is determined to be at risk:<br>• 1st degree relative with Diabetes<br>• Ethnic (South Asian, Japanese, Chinese, South American, Central American)<br>• Impaired fasting blood glucose (6.0 – 6.9) or impaired HbA1c (6.0-6.4) as documented in chart in the past year (up to 3 values)<br>• Hypertension or increase blood pressure or on medications for hypertension<br>• History of gestational diabetes<br>• Obese (BMI ≥30)<br>• High waist circumference<br>o South Asian, Japanese, Chinese, South American, and Central American Men: ≥90 cm<br>o All other Men: ≥102 cm<br>o South Asian, Japanese, Chinese, South American, and Central American Women: ≥80 cm<br>o All other Women: ≥88 cm<br>• Personal history of chronic kidney disease<br>• Personal history of cardiovascular disease	1,49,54

	• Personal history of hyperlipidemia<br>• Personal history of polycystic ovarian disease<br>On glucocorticoids, atypical antipsychotics or highly active antiretroviral therapy (HAART)	
Nutrition/Diet	Improvement in healthy eating habits as indicated by a decrease in score on the Starting the Conversation questionnaire (patients scoring ≥ 4 were eligible to make improvements in their eating habits).	1,42,43,51
Physical Activity	Target ≥ 150 minutes of vigorous activity per week OR<br>General Practice Physical Activity Questionnaire (GPPAQ) score equals "active"	2,39,48
Alcohol	At Risk Drinker:<br>WOMEN: >1 standard drinks on any one day OR >7 drinks/week OR ≥4 drinks at one time<br>MEN: ≥ 2 standard drinks on any one day for men OR ≥14 drinks men/week OR ≥5 drinks at one time	27,37
BMI	Value: Underweight: <18.5; Normal:18.5 – 24.9; Overweight: 25-29.9; Obese: ≥30<br>Calculation: kg/m² (weight in kilograms divided by height in meters squared)	29,31,32
Waist Circumference	Value:<br>Normal: < 102 cm for males or < 90 cm for S. Asian, Japanese, Chinese, ethic south and central American men; < 88 cm for females or < 80 cm for S. Asian, Japanese, Chinese, ethnic south and central American women<br>High: Males ≥ 102cm (40 inches) or ≥ 90 cm for S. Asian, Japanese, Chinese, ethnic south and central American men; Females ≥ 88 cm (35 inches) or ≥ 80 cm for S. Asian, Japanese, Chinese, ethnic south and central American women	49,50
Breast Cancer: Elevated Risk Factors	Personal history of BRCA1 or BRCA2 or other gene predisposing to a markedly elevated breast cancer risk<br>OR<br>Any case of<br>• Ovarian cancer OR<br>• Family member with BRCA1 or BRCA2 mutation OR<br>• Identified BRCA1 or BRCA2 mutation in a 1st degree relative and patient has not had genetic testing OR<br>• High risk ethnicity (Ashkenazi Jewish, Icelandic) AND personal or family history of breast and ovarian related cancers (breast, ovarian, male breast, pancreatic) OR<br>• One or two 1st degree relatives with invasive breast cancer	9-11,13
Breast Cancer Test and Review Frequency	All women ≥ 50 without elevated risk of Breast Cancer - Routine mammogram every 2 years	6-17
	All women 40-65 with elevated risk of Breast Cancer – Routine mammogram done every year	
Elevated Risk of colorectal Cancer (CRC)	Any of the following:<br>• ≥ 1 first degree relative with colorectal cancer diagnosis at ≥ 60 years of age (Alberta and Ontario ONLY) OR<br>• ≥ 1 first degree relative with colorectal cancer diagnosis at < 60 years of age OR	18-22,55

	• ≥ 2 first degree relatives with CRC diagnosis at any age OR<br>• ≥ 1 second degree relative with colorectal cancer diagnosis at < 50 years of age OR<br>• Personal history of inflammatory bowel disease (chronic ulcerative colitis or Crohn's disease) OR<br>• Personal history of familial adenomatous polyposis (FAP), Hereditary nonpolyposis colorectal cancer (HNPCC) or lynch syndrome (LS) OR<br>• Known carrier of mutation in LS gene OR<br>• Untested 1st degree relative of a LS mutation carrier	
CRC Testing and Review Frequency	If no risk factors, screening starts at age 50 as per one of the following:<br>• FOBT or FIT every 2 years;<br>• Sigmoidoscopy every 10 years; or<br>• Colonoscopy every 10 years if normal; if abnormal every 5 years.<br><br>If meet criteria for elevated risk:<br>• ≥ 1 first degree relative with colorectal cancer diagnosis at ≥ 60 years of age (Alberta and Ontario ONLY)<br>○ FIT every 2 years in AB (starting at age 40);<br>○ Colonoscopy every 5 years starting at 50 or 10 years younger than youngest diagnosis in ON<br>• ≥ 1 first degree relative with colorectal cancer diagnosis at < 60 years of age OR ≥ 2 first degree relatives with CRC diagnosis at any age OR<br>○ Colonoscopy starting at 50 or 10 years younger than youngest diagnosis in ON and NL;<br>○ Colonoscopy starting at 40 or 10 years younger than youngest diagnosis in AB<br>• ≥ 1 second degree relative with colorectal cancer diagnosis at < 50 years of age<br>○ Colonoscopy starting at 50 (repeat as indicated by findings)<br>• Personal history of inflammatory bowel disease (chronic ulcerative colitis or Crohn's disease), familial adenomatous polyposis (FAP), Hereditary nonpolyposis colorectal cancer (HNPCC), or lynch syndrome (LS)<br>○ Colonoscopy at discretion of GI<br>• Known carrier of mutation in LS gene OR untested first degree relative of a LS mutation carrier<br>○ Colonoscopy every 1-2 years starting at 20-25 or 2-5 years younger than youngest diagnosis if that diagnosis was made <25 years, whichever is earlier	
Cervical Cancer PAP Test and Review Frequency	If there is no abnormal cytology, no personal history of cervical cancer, no personal history of a hysterectomy with removal of the cervix AND:<br>• Patient is not immuno-compromised, pap test every 3 years<br>Patient is immune-compromised, pap test every year	23-26
Cervical cancer and immuno-compromised	For the purposes of cervical cancer screening, immuno-compromised is defined as meeting any of the following:<br>• Organ transplant recipient OR<br>• Chronic use of corticosteroids (i.e. a prescription ≥1 month) OR<br>• Prescription of an alkylating agent OR<br>• Prescription of an antimetabolite OR	25,56

	Prescription of a tumor necrosis factor (TNF) blocker	
ACE/ARB recommendation	If a patient does not have established cardiovascular disease or familial hypercholesterolemia, meets the following criteria, and is not already on an angiotensin converting enzyme (ACE) inhibitor or angiotensin receptor blocker (ARB), the patient should be referred to their primary care provider for an ACE/ARB discussion:<br>• Age >55 AND<br>• A personal history of at least one of the following: diabetes, coronary artery disease, cardiovascular disease, or peripheral vascular disease AND<br>• At least one of the following: hypertension, elevated total cholesterol (TC), low high density lipoprotein (HDL), currently smoking, or microalbuminuria	2,5

REFERENCES

1. Canadian Diabetes Association Clinical Practice Guidelines Expert C, Cheng AY. Canadian Diabetes Association 2013 clinical practice guidelines for the prevention and management of diabetes in Canada. *Can J Diabetes.* 2013;37 Suppl 1:S1-3.
2. Massimo F Piepoli AWH, Stefan Agewall, Christian Albus, Carlos Brotons, Alberico L Catpano, Marie-Therese Cooney, Ugo Corrà, Bernard Cosnys, Christi Deaton, Ian Graham, Michael Stephen Hall, F D Richard Hobbs, Maja-Lisa Løchen, Herbert Löllgen, Pedro Marques-Vidal, Joep Perk, Eva Prescott, Josep Redon, Dimitrios J Richter, Naveed Sattar, Yvo Smulders, Monica Tiberi, H Bart van der Worp, Ineke van Dis, W M Monique Verschuren, Simone Binno, ESC Scientific Document Group. 2016 European Guidelines on cardiovascular disease prevention in clinical practice: The Sixth Joint Task Force of the European Society of Cardiology and Other Societies on Cardiovascular Disease Prevention in Clinical Practice (constituted by representatives of 10 societies and by invited experts). *European Heart Journal.* 2016;37(29):2315-2381.
3. *Prevention and Management of Cardiovascular Disease Risk in Primary Care Clinical Practice Guideline. February 2015.*: Toward Optimized Practice;2015.
4. Lindsay P, Connor Gorber S, Joffres M, Birtwhistle R, McKay D, Cloutier L. Recommendations on screening for high blood pressure in Canadian adults. *Can Fam Physician.* 2013;59(9):927-933.
5. Nerenberg KA, Zarnke KB, Leung AA, et al. Hypertension Canada's 2018 Guidelines for Diagnosis, Risk Assessment, Prevention, and Treatment of Hypertension in Adults and Children. *Canadian Journal of Cardiology.* 2018;34(5):506-525.
6. Siu AL, USPSTF. Screening for Breast Cancer: U.S. Preventive Services Task Force Recommendation Statement. *Ann Intern Med.* 2016;164(4):279-296.
7. Tonelli M, Connor Gorber S, Joffres M, et al. Recommendations on screening for breast cancer in average-risk women aged 40-74 years. *CMAJ.* 2011;183(17):1991-2001.
8. Oeffinger KC, Fontham ET, Etzioni R, et al. Breast Cancer Screening for Women at Average Risk: 2015 Guideline Update From the American Cancer Society. *JAMA.* 2015;314(15):1599-1614.
9. *Indications for Use of Breast Magnetic Resonance Imaging (MRI) Guideline Summary.* Eastern Health;2011.
10. Agency BC. Breast. http://www.bccancer.bc.ca/health-professionals/clinical-resources/cancer-management-guidelines/breast/breast#Screening-Early-Detection. Published 2016. Accessed.
11. *Breast Cancer Screening Clinical Practice Guideline.* Toward Optimized Practice;2013.
12. *Magnetic resonance imaging for breast cancer screening, pre-operative assessment, and follow-up.* Alberta Health Services;2012.
13. Program KPMC. *Breast cancer screening clinical practice guideline.* Kaiser Permanente Medical Care Program;2014.
14. *Breast Magnetic Resonance Imaging (MRI) and High Risk Hereditary Breast Cancer Guideline Summary.* Eastern Health;2011.
15. *Magnetic resonance imaging screening of women at high risk for breast cancer.* Cancer Care Ontario;2007 (Updated 2012).
16. Daly MB, Pilarski R, Berry M, et al. NCCN Guidelines Insights: Genetic/Familial High-Risk Assessment: Breast and Ovarian, Version 2.2017. *J Natl Compr Canc Netw.* 2017;15(1):9-20.
17. Moyer VA, Force USPST. Risk assessment, genetic counseling, and genetic testing for BRCA-related cancer in women: U.S. Preventive Services Task Force recommendation statement. *Ann Intern Med.* 2014;160(4):271-281.
18. *Colorectal Cancer Screening Clinical Practice Guideline.* Kaiser Permanente Care Management Institute;2014.

19. *Colorectal Cancer Screening Clinical Practice Practice Guideline.* Toward Optimized Practice;2013.
20. Bacchus CM, Dunfield L, Gorber SC, et al. Recommendations on screening for colorectal cancer in primary care. *CMAJ.* 2016;188(5):340-348.
21. USPSTF, Bibbins-Domingo K, Grossman DC, et al. Screening for Colorectal Cancer: US Preventive Services Task Force Recommendation Statement. *JAMA.* 2016;315(23):2564-2575.
22. *Screening Guidelines - Colon Cancer.* Cancer Care Ontario;2016.
23. Saslow D, Solomon D, Lawson HW, et al. American Cancer Society, American Society for Colposcopy and Cervical Pathology, and American Society for Clinical Pathology screening guidelines for the prevention and early detection of cervical cancer. *CA Cancer J Clin.* 2012;62(3):147-172.
24. *Ontario Cervical Screening Guidelines Summary.* Cancer Care Ontario;2016.
25. Dickinson J, Tsakonas E, Conner Gorber S, et al. Recommendations on screening for cervical cancer. *CMAJ.* 2013;185(1):35-45.
26. Moyer VA, Force USPST. Screening for cervical cancer: U.S. Preventive Services Task Force recommendation statement. *Ann Intern Med.* 2012;156(12):880-891, W312.
27. *Cardiovascular disease: risk assessment and reduction, including lipid modification.* National Institute for Health and Care Excellence;2014.
28. Practice TO. Prevention and Risk Management of Cardiovascular Disease Risk in Primary Care Clinical Practice Guideline. 2015.
29. Jensen MD, Ryan DH, Apovian CM, et al. 2013 AHA/ACC/TOS guideline for the management of overweight and obesity in adults: a report of the American College of Cardiology/American Heart Association Task Force on Practice Guidelines and The Obesity Society. *Circulation.* 2014;129(25 Suppl 2):S102-138.
30. *Prevention and management of obesity for adults.* Institute for Clinical Systems Improvement;2013.
31. Moyer VA, Force USPST. Screening for and management of obesity in adults: U.S. Preventive Services Task Force recommendation statement. *Ann Intern Med.* 2012;157(5):373-378.
32. Paula Brauer SCG, Elizabeth Shaw, Harminder Singh, Neil Bell, Amanda R.E. Shane, Alejandra Jaramillo, Marcello Tonelli and Canadian Task Force on Preventive Health Care. Recommendations for prevention of weight gain and use of behavioural and pharmacologic interventions to manage overweight and obesity in adults in primary care. *2015.*187(3):184-195.
33. Runowicz CD, Leach CR, Henry NL, et al. American Cancer Society/American Society of Clinical Oncology Breast Cancer Survivorship Care Guideline. *CA Cancer J Clin.* 2016;66(1):43-73.
34. CAN-ADAPTT. Canadian Smoking Cessation Clinical Practice Guideline. Toronto, Canada: Canadian Action Network for the Advancement, Dissemination, and Adoption of Practice-informed Tobacco Treatment, Centre for Addiiction and Mental Health. 2011.
35. *Tobacco screening and treatment for adult cancer patients.* Alberta Health Services;2016.
36. *Diagnosis and Management of Colorectal Cancer. A National Clinical Guideline.* Scottish Intercollegiate Guidelines Network (SIGN);2016.
37. *Problem Drinking.* BC Guidelines;2013.
38. *Physical activity: exercise referral schemes.* National Institute for Health and Care Excellence;2014.
39. *Canadian Physical Activity Guidelines - Adults 18-64 Years.* Canadian Society for Exercise Physiology;2011.

40. *Canadian Physical Activity Guidelines. Older Adults - 65 Years & Older.* Canadian Society for Exercise Physiology;2011.
41. *Nutrition Guidelines - Cardiovascular Care - Hypertension.* Alberta Health Services;2016.
42. *Nutrition Guideline - Cardiovascular Care - Heart Healthy.* Alberta Health Services;2016.
43. *Nutrition Guideline - Adult Weight Management.* Alberta Health Services;2012.
44. Howell D. KH, Esplen M.J., Hack T., Hamel M., Howes J., Jones J., Li M., Manii D., McLeod D., Mayer C., Sellick S., Riahizadeh S., Noroozi H., & Ali M. *Pan-Canadian Practice Guideline: Screening, Assessment and Management of Psychosocial Distress, Depression and Anxiety in Adults with Cancer.* 2015.
45. *Depression in Adults: Recognition and Management.* National Institute for Health and Care Excellence;2009.
46. *Health Care Guideline: Adult Depression in Primary Care.* Institute for Clinical Systems Improvement;2016.
47. Siu AL, USPSTF, Bibbins-Domingo K, et al. Screening for Depression in Adults: US Preventive Services Task Force Recommendation Statement. *JAMA.* 2016;315(4):380-387.
48. *General Practise Physical Activity Questionnaire.* Physical Activity Policy, Health Improvement Directorate;2009.
49. *Nutrition Guideline - Diabetes.* Alberta Health Services;2016.
50. Diabetes Canada Clinical Practice Guidelines Expert C, Wharton S, Pedersen SD, Lau DCW, Sharma AM. Weight Management in Diabetes. *Can J Diabetes.* 2018;42 Suppl 1:S124-S129.
51. Paxton AE, Strycker LA, Toobert DJ, Ammerman AS, Glasgow RE. Starting The Conversation: Performance of a Brief Dietary Assessment and Intervention Tool for Health Professionals. *American Journal of Preventive Medicine.* 2011;40(1):67-71.
52. Association AD. Standards of medical care in diabetes–2015. 2015;Suppl 1:S1-S90.
53. Pottie K, Jaramillo A, Lewin G, et al. Recommendations on screening for type 2 diabetes in adults. *CMAJ.* 2012;184(15):1687-1696.
54. Siu AL, USPSTF. Screening for Abnormal Blood Glucose and Type 2 Diabetes Mellitus: U.S. Preventive Services Task Force Recommendation Statement. *Ann Intern Med.* 2015;163(11):861-868.
55. Provenzale D, Gupta S, Ahnen DJ, et al. Genetic/Familial High-Risk Assessment: Colorectal Version 1.2016, NCCN Clinical Practice Guidelines in Oncology. *J Natl Compr Canc Netw.* 2016;14(8):1010-1030.
56. *Cervical screening guidelines.* Eastern Health;2011.
