# Supplementary material for: Opportunities to improve quality of care for cancer survivors in primary care: findings from the BETTER WISE study
Source: Support Care Cancer. 2023 Jun 30;31(7):430. doi: 10.1007/s00520-023-07883-4 (PMC10313555; doi:10.1007/s00520-023-07883-4)
Supplement: Supplementary File 5 — File Name: Lofters - Appendix E - BETTER WISE Cancer surveillance Composite Index. File format: PDF. Title and description: The BETTER WISE Project Primary Outcome Source Document: Cancer Surveillance Composite Index [file 520_2023_7883_MOESM5_ESM.pdf]

Article Title: Opportunities to Improve Quality of Care for Cancer Survivors in Primary Care: Findings from the BETTER WISE Study

Journal Name: Journal of Cancer Survivorship

Author Names: Aisha Lofters, Ielaf Khalil, Melissa Shea-Budgell, Christopher Meaney, Nicolette Sopcak, Carolina Fernandes, Rahim Moineddin, Denise Campbell-Scherer, Kris Aubrey-Bassler, Donna Patricia Manca, Eva Grunfeld.

Corresponding Author: Dr. Aisha Lofters

Corresponding Author Affiliations:

1. Department of Family and Community Medicine, University of Toronto, 500 University Ave, Toronto, Ontario M5G 1V7, Canada
2. Peter Gilgan Centre for Women's Cancers, Women's College Hospital, 76 Grenville St, Toronto, ON M5S 1B2

Corresponding Author Email: aisha.lofters@utoronto.ca

Building on Existing Tools to Improve Cancer and Chronic Disease Prevention and Screening in Primary Care for Wellness of Cancer Survivors and Patients

The BETTER WISE Project

Primary Outcome Source Document:

Cancer Surveillance Composite Index

April 6, 2021

SECTION A: GENERAL INFORMATION 3

SECTION B: CDPS Composite Index Table 4

Section B.1 BETTER WISE Main CDPS Composite Index Table 4

SECTION C: INDICATORS AND RANGES 6

References – TO BE UPDATED 8

SECTION A: GENERAL INFORMATION

Purpose of this document

This document was prepared from the recommendations made by the Clinical Working Group for items regarding cancer surveillance for breast, colorectal, and prostate cancer survivors. Information on the criteria identified for each item or "criteria for eligibility (E)" AND "criteria for achievement (A)" listed within is to be collected and analyzed for the project's primary analysis. All recommendations were based on 2017 guidelines to improve quality in family practice.

This document contains the **Cancer Surveillance Composite Index Table** which define the referrals/actions patients are deemed eligible (E) to achieve based on assessments at the baseline evaluation and the criteria that must be met in order for a patient to have achieved (A) each referral/action they have been determined to be eligible for. All CCDPS items identified by the Clinical Working Group have been compiled to create the BETTER WISE Main Cancer Surveillance Composite Index. These items include process, referral/treatment, and target/change actions. As there is an association between process measures, referral/treatment actions, and patient outcomes (target/change outcomes), items encompassing these three areas have been included in the Main Composite Index.

- The **BETTER WISE Main Cancer Surveillance Composite Index Table (Section B.1),** defines the criteria used to calculate the main outcome cancer surveillance composite index for the project. The items described in this table allow for a calculation of the proportion change in unachieved cancer surveillance items for each patient and includes *process, referral/treatment, and target/change actions*. This composite index is calculated at follow-up (12 and 24 months, where applicable). The eligible actions are determined at baseline using the "criteria for eligibility (E)" and at each follow-up evaluation the achievement of the eligible actions is assessed using the "criteria for achievement (A)".
- Where the term Health Professional appears in this document it refers to Primary Care Practitioner, Nurse, Nurse Practitioner, Prevention Practitioner, Dietician, Nutritionist, Pharmacist, Clinical Assistant.

- **Indicators and Ranges (Section C)**, which specify the targets/ranges used to determine a patient's eligibility (E) for and achievement (A) of the referrals/actions included in the composite index at each evaluation time point (i.e. baseline, 12 month, and 24 month, where applicable).

This document was created by Dr. Eva Grunfeld, Dr. Donna Manca, Dr. Kris Aubrey-Bassler, Dr. Denise Campbell-Scherer, Dr. Aisha Lofters, Ms. Melissa Shea-Budgell, Dr. Rahim Moineddin, Christopher Meaney, Dr. Paul Kruger, Dr. Nicolette Sopcak, and Ms. Carolina Fernandes from the BETTER WISE Project team.

SECTION B: CDPS COMPOSITE INDEX TABLE

SECTION B.1 BETTER WISE MAIN CDPS COMPOSITE INDEX TABLE

Defines the criteria used to calculate the main composite index for the project, which includes *monitoring and screening actions*. This composite index is calculated at each follow-up evaluation (12 and 24 month, where applicable). The eligible actions are determined at baseline using the "criteria for eligibility (E)" and at each evaluation, the achievement of the eligible actions is assessed using the "criteria for achievement (A)". Items followed by a "*", "**", "***" are process outcome, referral/treatment outcome or target/change outcomes, respectively.

#	Referral/Action	Criteria for Eligibility (E) obtained and set at baseline	Criteria for Achievement (A) at follow-up visits every 6 months	References
1	Surveillance PSA*	All men 40-65 with a personal history of prostate cancer AND risk of recurrence is low, intermediate or unknown as documented in the chart that have not had a surveillance prostate specific antigen (PSA) test in the last year.	Surveillance PSA complete	1,2
		All men 40-65 with a personal history of prostate cancer AND risk of recurrence is high as documented in the chart that have not had a surveillance prostate specific antigen (PSA) test in the last 6 months or it is recommended by the oncologist that a PSA be completed in the last 6 months (as documented in the chart).	Surveillance PSA complete	
2	Surveillance colonoscopy*	All patients 40-65 with a personal history of CRC that have not completed a surveillance colonoscopy in the last 5 years if time from surgery/diagnosis ≥1 year.	Surveillance colonoscopy complete	3-7
3	Surveillance CEA*	All patients 40-65 with a personal history of CRC that have not completed a surveillance carcinoembryonic antigen (CEA) test in the last 6 months if time from diagnosis/surgery ≤ 5 years ago.	Surveillance CEA complete	3,8
4	Surveillance CT scan*	All patients 40-65 with a personal history of CRC that have not completed a surveillance computed tomography (CT) scan of the abdomen and chest in the last year AND time from diagnosis/surgery ≤ 3 years.	Surveillance CT scan complete	9
5	Breast cancer surveillance*	All women 40-65 with a personal history of breast cancer AND with breast tissue present AND without elevated risk of recurrence that have not had a surveillance mammogram completed in the last year.	Surveillance mammography complete	9-12
		All women 40-65 with a personal history of breast cancer AND with breast tissue present AND with elevated risk for breast cancer recurrence (see Section C) that have not had surveillance mammogram completed in the last year.	Surveillance mammography complete	
		All women 40-65 with a personal history of breast cancer AND with breast tissue present AND with elevated risk for breast cancer recurrence (see Section C) that have not had surveillance magnetic resonance imaging (MRI) completed in the last year.	Surveillance MRI complete	

6	Bone density screen*	All women 40-65 without a personal history of osteoporosis AND not receiving osteoporosis treatment AND with a personal history of breast cancer AND post-menopausal who have not completed bone density screening in the last 3 years.	Bone density screen complete	[2,9,11]
		All women 40-65 without a personal history of osteoporosis AND not receiving osteoporosis treatment AND with a personal history of breast cancer AND pre-menopausal on adjuvant endocrine medication (see Section C) who have not completed bone density screening in the last 3 years.	Bone density screen complete	
		All men 40-65 without a personal history of osteoporosis AND not receiving osteoporosis treatment AND with a personal history of prostate cancer AND on androgen deprivation therapy (ADT; see Section C) who have not completed bone density screening in the last 3 years.	Bone density screen complete	
7	Follow-up care plan*	All men 40-65 with a personal history of prostate cancer AND who do not have a follow-up care plan or equivalent (see Section C) documented on chart.	Follow-up care plan documented in chart	[4,13-15]
		All women 40-65 with a personal history of breast cancer AND who do not have a follow-up care plan or equivalent (see Section C) documented on chart.	Follow-up care plan documented in chart	
		All patients 40-65 with a personal history of colorectal cancer AND who do not have a follow-up care plan or equivalent (see Section C) documented on chart.	Follow-up care plan documented in chart	
8	Distress score improve*	All patients 40-65 with a personal history of breast or colorectal or prostate cancer AND who indicate a level of distress ≥ 4 on the Distress Thermometer	Improvement in distress score (i.e. decrease in distress score) since previous visit	[16]
9	Long-term effects and symptom management confidence improve*	All patients 40-65 with a personal history of breast or colorectal or prostate cancer AND who indicate a confidence level <7 in their ability to manage the long-term effects and symptoms resulting from their cancer treatment.	Improvement in confidence to manage their long-term effects and symptoms (i.e. increase in confidence) since last visit	[8,14,15]

SECTION C: INDICATORS AND RANGES

Domain	Target/Range	References
Breast Cancer Recurrence: Elevated Risk Factors	Any case of the following:<br>• ≥2 first or second-degree relatives with breast or ovarian cancer OR<br>• 1 first or second-degree relative with breast cancer < 50 (premenopausal) OR<br>• Family history of both breast and ovarian cancer OR<br>• ≥1 first or second-degree relatives with 2 cancers (breast and ovarian cancer or 2 independent breast cancers) OR<br>• Male relatives with cancer OR<br>• Women with cancer-predisposing mutations in either BRCA1 or BRCA2 OR<br>• Sister, mother, or daughter of a woman with a BRCA mutation OR<br>• Mutations in the TP53 gene (Li-Fraumei syndrome) and the PTEN gene (Cowden and Bannayan-Riley-Ruvalcaba syndromes) OR<br>• Women with Hodgkin disease treated with mantle field radiation treatment OR<br>• Women previously diagnosed with lobular neoplasia (lobular carcinoma in situ or atypical lobular hyperplasia) OR<br>• Women with high mammographic density	17
Breast Cancer Surveillance and Review Frequency	All women 40-65 with a personal history of breast cancer AND without elevated risk of recurrence - Routine mammogram every year<br>All women 40-65 with a personal history of breast cancer AND with elevated risk of recurrence - Routine mammogram AND MRI every year	9-12
SERMs recommendation	Women 40-65 should be encouraged to report any abnormal vaginal bleeding to their primary care provider if the use any of the following SERMs:<br>• Tamoxifen (Apo-Tamox, Gen-Tamoxifen, Nolvadex-D, Novo-Tamoxifen, Tamofen)<br>• Raloxifene (Evista, Act Raloxifene, Apo-Raloxifene, Novo-Raloxifene, Pms-raloxifene, Raloxifene, Raloxifene (Generic), Raloxifene Hydrochloride, Teva-raloxifene)<br>• Bazedoxifene (Duavive)	11,12
Bone density screen – Adjuvant endocrine medication	Women 40-65 with a personal history of breast cancer AND who are pre-menopausal and on adjuvant endocrine medication should complete bone density screening every 2-3 years.<br><br>The following are adjuvant endocrine mediations:<br>• Anastrozole (Arimidex)<br>• Buserelin Acetate (Suprefact)<br>• Exemestane (Aromasin)<br>• Goserelin Acetate (Zoladex)<br>• Letrozole (Femara)<br>• Leuprolide Acetate (Lupron, Eligard)<br>• Tamoxifen (Apo-Tamox, Gen-Tamoxifen, Nolvadex-D, Novo-Tamoxifen, Tamofen)	9
Bone density screen – Androgen deprivation therapy	Men 40-65 with a personal history of prostate cancer AND who are on androgen deprivation therapy (ADT) should complete bone density screening every 2-3 years.	1,2

(ADT)	The following are ADTs:<br>• Abiraterone Acetate (ZyJga)<br>• Bicalutamide (Casodex)<br>• Buserelin Acetate (Suprefact)<br>• Cyproterone Acetate (Androcur)<br>• Degarelix Acetate (Firmagon)<br>• Enzalutamide (Xtandi)<br>• Flutamide (Euflex)<br>• Goserelin Acetate (Zoladex)<br>• Histrelin Acetate (Vantas)<br>• Leuprolide Acetate (Lupron, Eligard)<br>• Nilutamide (Anandron)<br>• Triptorelin Pamoate (Trelsatr)	
Alcohol	At Risk Drinker for cancer survivors:<br>WOMEN with a personal history of breast and/or colorectal cancer: >0 standard drinks a week OR ≥4 drinks at one time<br>MEN with a personal history of prostate and/or colorectal cancer: >0 standard drinks a week OR ≥5 drinks at one time	[2,11]
Follow-up Care Plan definition	For all cancer survivors, the following items should be covered in a follow-up care plan or equivalent:<br>• History and physical examination schedule;<br>• Description of care coordination (i.e. who is most responsible for what care); and<br>• Monitoring of long-term side effects, symptoms of recurrence, and psychosocial well-being.<br><br>For breast cancer survivors, these additional items should be covered:<br>• Surveillance mammography at recommended intervals;<br>• DEXA scan q2-3 yrs;<br>• Annual monitoring of abnormal vaginal bleeding for women on SERMs;<br>• Endocrine therapy adherence assessment; and<br>• Calcium and vitamin D recommendations.<br><br>For colorectal cancer survivors, these additional items should be covered:<br>• Surveillance colonoscopy, CEA measurement, and follow-up imaging (CT) at recommended intervals.<br><br>For prostate cancer survivors, these additional items should be covered:<br>• Measurement of serum PSA at recommended intervals;<br>• Annual digital rectal exam (DRE);<br>• DEXA scan q2-3 yrs; and<br>• Calcium and vitamin D recommendations.	[1,7,12]

Legend: ADT = androgen deprivation therapy; CEA = carcinoembryonic antigen; CRC = Colorectal Cancer; CT = computed tomography; MRI = magnetic resonance imaging; PSA = prostate specific antigen; SERM = selective estrogen receptor modulator

References

1. *Physician Prostate Cancer Transfer of Care Sample Letter.* Alberta Health Services.
2. *Prostate Cancer.* Alberta Health Services;2015.
3. *Colon cancer: diagnosis, treatment and follow-up.* Belgian Health Care Knowledge (KCE);2014.
4. *Stages I and II Colorectal Cancer Surveillance - Clinical Practice Guidelines.* Alberta Health Services;2014.
5. *Follow-up and Surveillance of Colon Cancer Patients Treated with Curative Intent.* BC Cancer Agency;2018.
6. El-Shami K, Oeffinger KC, Erb NL, et al. American Cancer Society Colorectal Cancer Survivorship Care Guidelines. *CA Cancer J Clin.* 2015;65(6):428-455.
7. Steele SR, Chang GJ, Hendren S, et al. Practice Guideline for the Surveillance of Patients After Curative Treatment of Colon and Rectal Cancer. *Diseases of the Colon & Rectum.* 2015;58(8):713-725.
8. *Follow-up care, surveillance protocols and secondary prevention measures for survivors of colorectal cancer.* Cancer Care Ontario;2016.
9. Runowicz CD, Leach CR, Henry NL, et al. American Cancer Society/American Society of Clinical Oncology Breast Cancer Survivorship Care Guideline. *CA Cancer J Clin.* 2016;66(1):43-73.
10. *Breast cancer in women: diagnosis, treatment and follow-up.* Belgian Healthcare Knowledge Centre;2013.
11. *Follow-Up Care for Early-Stage Breast Cancer - Clinical Practice Guidelines.* Alberta Health Services;2015.
12. *Physician Breast Cancer Transfer of Care Sample Letter.* Alberta Health Services;2016.
13. *Patient Prostate Cancer Transfer of Care Sample Letter.* Alberta Health Services.
14. *Breast.* BC Cancer Care Agency;2014.
15. *Follow-up Care and Psychosocial Needs of Survivors of Prostate Cancer.* Cancer Care Ontario;2015.
16. *Screening for Distress, the 6th Vital Sign: A Guide to Implementing Best Practices in Person-Centred Care.* Canadian Partnership Against Cancer;2012.
17. *Genetic/Familial High-Risk Assessment: Breast and Ovarian.* National Comprehensive Cancer Network;2016.
